# 

**Published:** 1886-07

**Authors:** 


### SOCIETY MEETINGS.

### Pennsylvania Society.

Eighteenth Annual Meeting of the Pennsylvania State Dental Society, Tuesday, July 27th, 1886, at 10 o'clock A.M., continuing three days.

The following programme has been prepared:

ESSAYS.

1. Dr. Louis Jack, Philadelphia—Distinctions between the Indications of Hyperæsthesia and those of Disturbances of the Blood Vessels of the Dental Pulp.

2. Dr. H. Leffmann, Philadelphia—Notes on Adulterations and Defective Preparation of Dental Materials.

3. Dr. W. B. Miller, Altoona—Duplex Spring and other Matrices.

4. Dr. H. C. Register, Philadelphia—Compressed and Warm Air as a Germicide, Pain Obtunder, and otherwise Useful Agent in Dental Practice.

5. Dr. E H. Neall, Philadelphia—Review of the Causes of the Decline of Mechanical Dentistry.

6. Dr. S. E. Feltwell, Pittsburg—Abscess and its Treatment.

7. Dr. C. S. Beck, Wilkesbarre—Incidents of Practice.

8. Dr. J. C. M. Hamilton, Tyrone—The Dental Pulp and its Treatment.

9. Dr. J. A. Todd, Titusville—Are we improving?

10. Dr. S. B. Duckie, Chester—Cavity Lining.

11. Dr. L. Campbell, Slatington—Incidents of Practice.

12. Dr. W. E. Van Orsdel, Mercer—All sorts of Questions from Practice.

13. Dr. R. B. Cummins, Blairsville—A Study in Dentistry.

14. Dr. E. C. Blackburn, Hollidaysburg--Incidents of Practice.

The Examining Board will be prepared to meet candidates for examination and sign diplomas.

---

To the members of and delegates to the American Dental Association, and all dentists:

The undersigned has succeeded in making arrangements with the Trans-Continental and other lines of railway, so that, although the meeting of the Association is to be held at Niagara (its Ex. Com. having instructed the Chairman to call it there in the interests of harmony), the profession will still be able to avail themselves of the privilege of a visit to the Pacific coast at the low rates given to the Grand Army. An extension of the time for arriving at San Francisco has been granted, so that all desiring to attend the meeting at Niagara can do so and then have until August 17th to reach the Pacific coast. Tickets will have to be purchased during the month of July, previous to the 25th, but will be good going from July 3d to arrive at San Francisco on or before August 17 and good to return for 85 days from August 3d. Certificates and information will be supplied upon application to A. M. DUDLEY, Salem, Mass.

### The American Dental Association.

The committee of arrangements had hoped to give full and definite information in the July number of Journals in regard to all the details of the arrangements for the annual meeting of the Association to be held at Niagara, August 3d. But as the railroad rates thus far secured have not been as satisfactory as the committee yet hope to secure and are working for, they will issue a circular later to all members of the Association and to local societies as far as possible. All State and local societies which have adopted substantially the Code of Ethics of the American Dental Association will remember that they are entitled to one delegate for every five members. Such delegates must have credentials signed by the President and Secretary of the society which they represent. Those who are not members of the Association and who wish the circular will please drop a postal to the chairman of the committee to insure their getting it. The railroad rates as thus far secured on all leading lines are one and a third fare round trip, to be issued upon presentation of certificate. Definite information concerning this will be given in the circular if better terms and arrangements are not secured.

The hotel rates will be as follows: The International Hotel will receive dentists and their families at $3.00 per day. The Cataract at $4.00 per day. The Niagara, Prospect Park, and Atlantique, $2.00 per day, if rooms are applied for and secured in advance.

The Park Theatre, adjoining the International Hotel, has been secured as place of meeting. Do not be anxious about not receiving the circular. You will get it some time in July; but a few days delay in issuing the circular may mean a good deal of money saved to those attending the Association. For instance, the arrangements were not completed and circular issued until latter part of July last year. A month earlier it would have been *impossible* to have gotten the low rates finally secured. Remember we *promise* nothing better than we now publish, but will continue to work for more favorable terms.

J. N. Crouse, Chr. of Com. of Arr.

2231 Prairie Ave., Chicago.

Cincinnati, May 1, 1886.

The third annual meeting of the National Association of Dental Faculties will be held at Niagara Falls, Monday, August 2nd, at 2 P.M.

C. N. Pierce, President.
H. A. Smith, Secretary.

The Minnesota State Dental Association will meet at St. Paul, July 21st, 22d, and 23rd; and will hold its sessions in the State Capitol.

M. G. Jenison, Secretary.

### American Dental Association.

The Twenty-sixth Annual Meeting of the American Dental Association will be held at Niagara Falls, commencing at 10 A. M., on Tuesday, Aug. 3d, 1886.

Geo. H. Cushing, Secretary.

### National Association of Dental Examiners.

The annual meeting will be held at 11 A. M. on Monday, Aug. 2d, 1886, at Niagara Falls. It is hoped all State Boards will be represented.

Geo. H. Cushing, Secretary.

### Delegates.

The following named persons were appointed delegates to the American Dental Association from the Mississippi Valley Dental Society at the meeting in March last, viz.:

Drs. Chas. Welch, Wilmington, O.; J. R. Callahan, Hillsboro, O.; A. H. Beamer, Cynthiana, Ky.; J, W. Jay, Richmond, Ind.; C. I. Keely, A. T. Good, Hamilton, O; W. D. Kempton, N. S. Hoff, C. M. Wright, E. G. Betty, F. W. Sage, A. G. Rose, H. L. Moore, M. H. Fletcher, Cincinnati, O. It is to be hoped that every one of these appointees will be present at the meeting at Niagara.

### EDITORIAL.

### THE DENTAL REGISTER,

A MONTHLY MAGAZINE DEVOTED TO THE INTERESTS OF THE

### DENTAL PROFESSION.

*Terms Reduced to* $2.00 *Per Year. Single Copies,* 25 *Cents.*

### EDITED BY PROF. J. TAFT, D.D.S.

—AND—

### Published by SAM'L A. CROCKER & CO., Ohio Dental and Surgical Depot.

The volume commences in January and closes in December.

The REGISTER being issued monthly is always fresh, therefore Indispensable to the practitioner who desires to keep posted up with the new inventions and improvements which are daily developed. Neither expense nor effort will be spared to give value and variety to its contents. Articles will be illustrated with well executed engravings whenever they will add to the interest or assist to explanation.

Its extensive circulation and frequency of issue make it one of the BEST DENTAL ADVERTISING MEDIUMS in the United States.

All subscriptions must begin with January or July.

Local or special notices, five lines or less, will be inserted for $3 each insertion; over five lines, 50 cts. per line.

All advertising bills payable in advance, or at furthest, after the issue of the first number containing the advertisement. All transient advertisements to be paid for in advance.

☞CONTRIBUTIONS SOLICITED.

Exclusively Editorial Communications should be addressed to the Editor.

The latest number of the REGISTER may be ordered with or without other goods at any time, and all letters should be addressed to

SAM'L A. CROCKER & CO., Ohio Dental and Surgical Depot, Cincinnati, O.

